# Prediction of Dropout in a Randomized Controlled Trial of Adjunctive Light Treatment in Patients with Non-Seasonal Depression and Evening Chronotype

**DOI:** 10.3390/clockssleep4030029

**Published:** 2022-07-27

**Authors:** Joey W.Y. Chan, Shirley Xin Li, Steven Wai Ho Chau, Ngan Yin Chan, Jihui Zhang, Yun Kwok Wing

**Affiliations:** 1 Li Chiu Kong Family Sleep Assessment Unit, Department of Psychiatry, Faculty of Medicine, The Chinese University of Hong Kong, Shatin, Hong Kong, China; stevenwaihochau@cuhk.edu.hk (S.W.H.C.); rachel.chan@cuhk.edu.hk (N.Y.C.); jihui.zhang@cuhk.edu.hk (J.Z.); ykwing@cuhk.edu.hk (Y.K.W.); 2 Department of Psychology, The University of Hong Kong, Pokfulam, Hong Kong, China; shirleyx@hku.hk; 3 The State Key Laboratory of Brain and Cognitive Sciences, The University of Hong Kong, Pokfulam, Hong Kong, China

**Keywords:** light therapy, depressive disorder, attrition, dropout, eveningness

## Abstract

The current study examined the possible predictors of dropout during a five-week light treatment (LT) with a gradual advance protocol in 93 patients with unipolar non-seasonal depression and evening chronotypes by comparing their clinical characteristics and performing a logistic regression analysis. Nineteen out of ninety-three (20%) subjects (80% female, 46.5 ± 11.7 years old) dropped out during the 5-week light treatment. Treatment non-adherence (i.e., receiving LT for less than 80% of the prescribed duration) over the first treatment week predicted a five-fold increase in risk of dropout during light therapy (OR: 5.85, CI: 1.41–24.21) after controlling for potential confounders, including age, gender, treatment group, rise time at the baseline, patient expectation, and treatment-emergent adverse events. There is a need to incorporate strategies to enhance treatment adherence and retention in both research and clinical settings. Chinese clinical trial registry (ChiCTR-IOR-15006937).

## 1. Introduction

Depression is a common mental health problem and a leading cause of mental health-related burdens across the globe [1]. It is associated with a range of adverse outcomes, including reduced daytime functioning, a wide range of secondary disorders, and increased early mortality due to physical disorders and suicide [2]. Emerging evidence has shown that bright light therapy (BLT) could be an efficacious treatment for non-seasonal depression, either as a monotherapy or an adjunct therapy to antidepressants [3,4,5]. Several randomized controlled trials have demonstrated the efficacy of adjunctive bright light therapy in reducing depressive symptoms of non-seasonal depression in major depressive disorders (MDDs) [6,7,8]. A meta-analysis which included 23 randomized controlled trials (RCTs) demonstrated a beneficial effect of light therapy in patients with non-seasonal depression with a standardized mean difference (SMD) in depression score of −0.40 (95% CI −0.60 to −0.21) [3].

Previous research showed that light therapy was generally well-tolerated and that adverse effects were mild and transient [4]. However, wide variation in dropout rates in trials of adjunctive light therapy in non-seasonal depression were observed, varying from 13% in a study with patients of unipolar depression [7] to 44% [9] in studies involving bipolar depression patients. The mean attrition rate was 17% in a systematic review involving light therapy studies on patients with intrinsic circadian rhythm sleep disorders and neuropsychiatric disorders [10]. Not only do dropouts significantly hamper treatment effects [11], they may also influence study power and generalizability and cause potential bias, as the remaining participants might differ from the overall sample recruited at the baseline [12].

Furthermore, unipolar depression patients with concomitant eveningness (i.e., a circadian preference towards a later timing for rest and activity; individuals with such chronotypes also being referred to as ‘night owls’) may have a higher propensity to drop out in interventions (e.g., adjunctive light therapy), as they tend to have lower self-control [13], more lifestyle irregularity [14], higher impulsivity [15], and greater alcohol and substance use [16], which could all contribute to poorer treatment adherence and worse outcomes. Eveningness is common among patients with MDD, with a prevalence rate of around 20% [17]. Depressed patients with eveningness have been found to have poorer outcomes of depression, including higher non-remission rates and suicidality [17,18,19]. An improved understanding of the factors associated with these patients’ tendencies to drop out might shed light on how to improve subject retention and enhance therapeutic effects in this vulnerable group.

In the treatment of depression, demographics and types of treatment or medications have been implicated as predictors of dropout [20,21,22]. On the other hand, dropout in light therapy trials could also be related to a variety of factors, including patient expectations, treatment efficacy, and adverse events, but such reasons have been underreported in light treatment studies [10]. Light therapy was reported to have a quick onset of therapeutic effect, as early as 1 week [23]. However, in an antidepressant trial involving 1008 adults with MDD, a higher burden of side effects as early as 4 days post-treatment was associated with poorer treatment outcomes [24]. In a randomized controlled trial (RCT) of depression treatment involving 1646 Japanese adults with non-psychotic MDD, those who dropped out of treatment were found to be less adherent to treatment even at the first week of treatment [25]. Thus, the limited evidence showed that non-adherence to treatment in the early weeks may impact dropout rates and the treatment outcomes. However, there is a dearth of data focusing on treatment adherence and change in clinical outcomes in the initial weeks which might be informative for predicting dropout in the course of light treatment. The current study aimed to investigate baseline demographic data, treatment expectations, sleep patterns, and clinical differences as potential predictors of dropout in a 5-week trial of light treatment with a gradual advance protocol and to explore the possible role of side effects, treatment adherence, and change in clinical outcomes in the first week as early predictors of dropout. Adherence in this study was defined as receiving 80% or more of the prescribed duration of light therapy in a week; this amount was arbitrarily chosen, as a previous study on seasonal affective disorder having found that adherence to the prescribed duration of exposure averaged 83% among the completers of four weeks of light treatment [26]. Dropout was defined as premature termination of treatment prior to the 5-week endpoint.

## 2. Results

### 2.1. Baseline Characteristics

**Figure 1:**
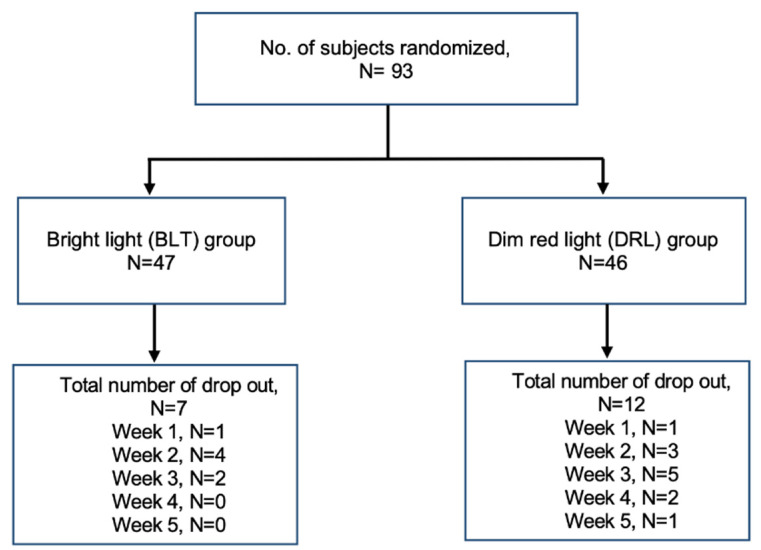
Subject flow diagram.

A total of 93 participants were included in the analysis. The mean age was 46.5 ± 11.7 (years ± SD) and 80% were female. Nineteen out of ninety-three (20%) of the participants dropped out during the five weeks of light treatment (Figure 1, subject flow diagram). There were no differences in age, gender, education level, living status, monthly income, and season of enrollment to the study. The expectation towards the effectiveness of treatment in both groups was also similar. Sleep diary data showed later rising times in the Dropout group than in the Completer group (11:12 h ± 02:27 vs. 10:13 h ± 01:54 (time ± SD, *p* = 0.066)), but there were no differences in sleep duration or sleep midpoint. The duration of depressive illness appeared to be longer in the Dropout group but was not statistically significant (17.0 ± 13.9 vs. 12.9 ± 10.1 (years ± SD, *p* = 0.19)). The baseline scores for 17-HDS, atypical symptom score for SIGH, HAMA, HADS, ISI, SF-36, CFS, YMRS, and MEQ were not significantly different between the two groups (Table 1).

### 2.2. Treatment Characteristics

Seven out of forty-seven subjects (15%) and 12 out of 46 subjects (26%) dropped out in the bright light therapy group and dim red light group, respectively (*p* = 0.41). Over 70% of subjects were put on psychotropic medications. There was no difference in the prescription of psychotropic medications between the two groups (Table 2).

### 2.3. Early Treatment Adherence and Clinical Outcome Changes

As two subjects (one from the bright light therapy group and one from the dim red light group) dropped out before the first-week assessment, they were excluded from the analysis of early treatment adherence and change in clinical outcomes. Both prescribed and recorded light therapy start times appeared to be later in the Dropout group but were not significantly different. However, the total weekly duration of light therapy in the first week was significantly higher in the Completer than in the Dropout group (174 ± 54 vs. 110 ± 82 (minutes ± SD, *p* = 0.007)). The percentage of participants who received LT treatment for more than 80% of the prescribed duration was also higher for the completers than for the dropouts, 62% vs. 23% (*p* = 0.004), respectively. There was no difference in the incidence of treatment-emergent adverse events at week 1 between the dropouts and the completers. Regarding clinical outcomes, both groups showed a trend towards improvement with a reduction in mean scores for 17-HDS, HAMA, HADS, BSSI, and CFS, without significant group differences. YMRS score was slightly higher in the Dropout group as compared to the Completer group, but the magnitude of change (−0.2 ± 1.8 vs. 0.8 ± 2.1) was unlikely to be clinically significant. The overall rate for missing data was 5% (Table 3).

### 2.4. Predictors for Dropout

In the logistic regression, Model 1 included the variables with *p* < 0.1 in the univariate analysis (i.e., the change of YMRS score in week 1 and rise time at the baseline). The categorical variable of “getting less than 80% duration of light therapy in the first week” was chosen over the continuous variable of “the weekly total duration of light therapy performed” to allow easier identification of at-risk individuals in the clinical setting. Those who received less than 80% of the total duration of light therapy prescribed in the first week had a 4.6-fold higher risk of dropout during weeks 2 to 5 of the light therapy treatment program (OR: 4.69, CI: 1.32–16.66, *p* = 0.017). Model 2 additionally adjusted for age, gender, light treatment, patient expectation, and TEAE at the first week of treatment and showed similar results (OR: 5.85, CI: 1.41–24.21, *p* = 0.015) (Table 4).

## 3. Discussion

This study aimed to examine the effect of early treatment adherence and changes in clinical outcomes on the risk of dropout during a light treatment intervention. The results of our study showed that the overall dropout rate during the intervention phase was 20% (19 out of 93). Those subjects who received less than 80% of the total prescribed duration of light therapy in the first week had a five-fold increased risk of dropout during the course of light treatment and the risk was independent of other confounding variables, including age, gender, type of light treatment, rise time at baseline, patient expectation, and treatment-emergent adverse events.

### 3.1. Dropout Rate

The overall dropout rate of 20% in our study was comparable to the overall rate of dropout in mental health treatment—around 17–19% [27]. The mean attrition rate during the light treatment phase was 17% across studies of intrinsic circadian rhythm sleep disorders and neuropsychiatric illness [10]. The 20% rate for our study is slightly higher than those of other randomized controlled trials that focused on adult patients with non-seasonal MDD in the out-patient setting, which reported 9 and 13%, respectively [6,7]. This could be attributable to an exclusive evening-type sample characterized by more sedentary activity and less moderate-to-vigorous physical activity [28], unhealthy eating habits [29], lower self-control [13], and an overall more irregular lifestyle [14], hence greater difficulty in engaging in treatment. This suggests that for this particular group of patients, better strategies should be developed to reduce dropout and enhance treatment adherence. One might expect the attrition rate to be higher in the control group than in the intervention group. The dropout rate in this trial was numerically higher in the DRL group than the BLT group (26% vs. 15%) but did not reach statistical significance. This is consistent with the findings of 14% and 8% dropout in the intervention group versus the control group for light therapy studies in a systematic review [10]. Similar to another study focusing on adherence in light therapy, patient expectations were found not to be related to dropout [26]. The expectation score also did not differ between the BLT group and the DRL group in this trial [8]. There was a significant difference in the change of YMRS from baseline to week 1 between the bright light therapy (BLT) and dim red light (DRL) groups (−0.2 ± 1.8 vs. 0.8 ± 2.1, *p* = 0.046). None of the subjects had reached the cut-off of 12, which was the general cut-off for hypomania [30]. This suggested that bright light therapy is a safe antidepressant treatment with respect to hypomanic swings in depressed patients with eveningness.

### 3.2. Monitoring of Adherence

Our study highlighted the importance of adherence to light treatment in the initial week of intervention. However, monitoring adherence to light therapy remains a clinical challenge. Some studies have used self-reported treatment logs to capture adherence information [7,8,31]. Nonetheless, a pilot study focused on the adherence of bright light treatment in patients with seasonal affective disorder found no correlation between patient self-reports and the objectively measured durations of light box use objectively measured by a built-in meter [26]. This implies that a more objective method might be needed in future light therapy studies to monitor adherence. In this study, we defined compliance based on the duration of light therapy undergone [8]. A light data logger (HOBO U9 light on/off logger) was used in one study on bipolar depression to confirm appropriate, missed, or identified ill-timed light therapy sessions [31]. The light sensor on the actiwatch has been used to capture continuous light data during and around interventions, but there could be a potential problem in measuring light exposure at the wrist, as it is at a distance from the cornea and might be covered by clothes in winter months [32]. Figueiro et al. found that a light sensor placed as a pendant around the neck or pinned on the torso yielded similar photopic illuminance data as compared to a light sensor near the cornea [32]. Such measurements could be potentially useful as objective measurements of light exposure and serve as proxies for bright light treatment adherence.

### 3.3. Strategies to Enhance Adherence

In patients receiving antidepressant treatment, the systematic review by Pampallona et al. found a variety of interventions (for example, patient education and medication clinics) that have been used to enhance adherence to medications. Although the heterogeneity of the studies precluded a solid conclusion, the evidence consistently indicated that adherence to treatment can be increased through interventions [33]. Different approaches to adherence management for light therapy have been used, including supervision, education, reminders, environmental modification, etc. [10], but there is a paucity of data that would allow the systematic evaluation of their efficacy in improving adherence. In terms of optimizing treatment environments and support, BLT has been used an adjunct treatment with antidepressants in hospitalized patients with depression and was shown to hasten the effect of antidepressants [34,35]. The in-patient setting could be a supportive environment in which to initiate light therapy and consolidate its implementation. A recent study that tested the use of BLT for depressive symptoms in an acute psychiatric in-patient unit found a dropout rate of 9% and an adherence rate to daily BLT of 94%, even though participants were allowed to skip any session [36]. This study suggests that it is feasible to start BLT in acute psychiatric units to enhance early treatment adherence, although it is unclear whether the benefits could be extended after a patient is discharged from the hospital, as BLT might require several weeks for the treatment of depressive symptoms [7,31]. Further study is needed to provide confirmatory evidence that initiating BLT in acute in-patient units has greater benefits than in the out-patient setting.

Cognitive behavioral therapy (CBT) has also been used to enhance medication adherence in patients with major depressive disorder [37]. There is currently no trial evaluating whether CBT could enhance adherence to BLT and retention in treatment in patients with depression. There have been several trials that investigated the efficacy of a combination of CBT with BLT for young patients with delayed sleep phase. Gradisar et al. evaluated the use of combined CBT and concurrent BLT for adolescents with delayed sleep phase disorder (DSPD) and found that the combination treatment resulted in a greater improvement in sleep parameters, with more sustained effects in the group with CBT. There was a numerically lower rate of dropout of 11% (3 out of 26) in the CBT group and of 26% (6 out of 23) in the waitlist group, though characteristics of dropout were not described [38]. On the other hand, Danielsson et al. found an overall rate of protocol failure because of “never use the light box” in 16% (19 out 57) patients with delayed sleep phase disorder in a study of 4-week CBT following either 2 weeks of BLT or the control condition; however, the impact of CBT on BLT could not be evaluated, as BLT was completed before CBT [39]. Interestingly, in a larger trial involving 102 adolescents who complained of delayed sleep, the combination of light flash therapy plus CBT resulted in a seven-fold greater increase in bedtime compliance than that observed in the group with sham plus CBT [40]. This suggests that, reciprocally, BLT might also improve adherence to prescribed sleep schedules in CBT.

As the use of light treatment represents a behavioral change and often involves lifestyle modification (e.g., an earlier rise time, less use of electronic devices at night, etc.), motivational interviewing (MI) could be another strategy to enhance adherence to treatment. MI is a person-centered, goal-oriented method of communication for eliciting and strengthening intrinsic motivation for positive change [41]. MI has been evaluated for reducing depressive symptoms and was found to improve the trajectory of depression and increase receipt of antidepressants in the primary care setting [42]. It has also been used in patients with depression and comorbid substance use disorder to reduce substance use [43,44]. MI has been employed as a component in cognitive behavioral therapy for insomnia in patients with bipolar disorder (CBTI-BD) [45]. The overall treatment package yielded significant results in terms of reducing the number of days in a mood episode. The dropout rate after randomization, however, did not differ between the CBT-BD group and the control group (20% vs. 21%, respectively), albeit the component of MI was not separately evaluated in this trial. A combination of BLT with or without MI/CBT has not been tested in patients with depression; future trials may consider integrating MI techniques or using CBT as an adjunct to BLT to further improve adherence and outcomes.

### 3.4. Limitations

This was a prospective study that involved a well-defined patient group with unipolar depression and evening preference with weekly follow-up on the clinical outcomes during the intervention phase which allowed us to capture information about early changes in symptoms and adherence with respect to dropout. However, the measurement of adherence was subjective and actual treatment durations may have been overestimated. Future trials using objective measurements (e.g., measurements obtained by light-logging devices) may provide more accurate records. Secondly, the subjects included in this study were patients with unipolar nonseasonal depression and exclusively evening chronotypes, which might limit the generalizability of the results to other patients.

## 4. Materials and Methods

### 4.1. Participants

The current study analyzed the data collected in a randomized controlled trial (RCT) of adjunctive bright light therapy in patients with non-seasonal depression and concomitant eveningness. The detailed study procedure has been reported elsewhere [8,46]. In sum, 93 adult patients with a diagnosis of non-seasonal unipolar depression were recruited. They were assessed using the Mini-International Neuropsychiatric Interview (MINI) [47], the seasonal pattern specifier of DSM-V [48], and the 17-Item Hamilton Depression Score (17-HDS) component of the Structured Interview Guide for the Hamilton Depression Rating Scale with Atypical Depression Supplement (SIGH-ADS) [49]. The inclusion criteria for this study included: (i) Chinese subjects aged 18–65 years old; (ii) being capable of giving informed consent; (iii) meeting the diagnostic criteria of MDD according to MINI; (iv) scoring at least 14 on the 17-HDS; and (vi) scoring 41 or less in the Morningness–Eveningness Questionnaire (i.e., having an evening chronotype) [50]. Subjects with the conditions listed below were excluded from the study: (i) MDD fulfilling the seasonal pattern specifier of the DSM-V; (ii) a current diagnosis of substance abuse or dependence; a current or past history of manic or hypomanic episodes, schizophrenia, personality disorder, mental retardation, or organic mental disorder; (iii) significant suicidal risk in the opinion of the investigator, or a moderate or higher level of suicidality as assessed by the Suicidality Module of MINI, or having made a suicide attempt in the past 3 months; (iv) a history of light-induced migraine/epilepsy; (v) current use of photosensitizing medications; (vi) presence of eye disease, e.g., retinal blindness, severe cataracts, glaucoma; (vii) current therapy with drugs that could potentially interfere with circadian rhythms, i.e., lithium, exogenous melatonin, melatonergic antidepressants within past 3 months; (viii) shift worker; (ix) trans-meridian flight in the past 3 months or during the study; (x) significant medical condition/hearing impairment/speech deficit which might lead to incapability of completing the clinical interview. The trial was registered with the Chinese clinical trial registry (ChiCTR-IOR-15006937).

### 4.2. Intervention

Eligible participants were randomized to five weeks of either the active intervention group, which received 10,000 lux bright white light therapy (NatureBright Company, Irvine, CA, USA) (BLT group), or the placebo group, which received 50 lux dim red light (DRL group). Both groups received light therapy at home for 30 min a day; the initial timing of the light therapy was set at the habitual wake time, which was determined by the 1-week sleep diary prior to the baseline session. A prescriber reviewed the treatment log and sleep diary at each treatment week to determine the timing of the light therapy according to a gradual advance protocol: if the participant received adequate appropriately timed light therapy over the past week (defined as more than 50% of the total weekly duration of light therapy received, with timing overlapped with or earlier than the prescribed time of light therapy), the timing of the light therapy would be advanced for 30 min. On the other hand, if the participant was not able to achieve adequate appropriately timed treatment, the timing of the light therapy would be kept the same in the following week [8]. Each participant was prescribed a start time for light therapy by a psychiatrist, and they also recorded the actual light therapy start and end time in a treatment log. They were followed up weekly during the five-week light therapy. All participants continued their usual psychiatric treatment.

### 4.3. Outcomes

At the baseline, demographic, sleep, and clinical characteristics of the participants were collected. They were asked to rate their expectation towards the effectiveness of light therapy in improving their mood symptoms on a Likert Scale from 1 (not at all) to 100 (very much) prior to the start of treatment. In each of the follow-ups, participants were assessed for their clinical outcomes and side effects and returned their completed sleep dairies. The incidence of a treatment-emergent adverse event (TEAE) was defined as a change from baseline to at least moderate severity at follow-up. A range of clinical outcomes were measured weekly during the intervention, including the 17-HDS [49], Hamilton Anxiety Scale (HAM-A) [51], Hospital Anxiety and Depression Scale (HADS) [52], Insomnia Severity Index (ISI) [53], Beck’s Scale for Suicide Ideation (BSSI) [54], Chalder Fatigue Scale (CFS) [55], and quality of life assessed by the Short-Form 36-Item Health Survey (SF36) [56]. The self-reported questionnaires were translated and validated in Chinese [57,58,59,60,61]. The internal consistency (Cronbach’s alpha) of the results for the 17-HDS, HAMA, HADS, ISI, BSSI, CFS, and SF36 were 0.81, 0.92, 0.79, 0.87, 0.67, 0.91, and 0.91, respectively. The primary outcomes of the RCT were the cumulative rate of achieving remission of depression (defined as a 17-HDS score of 7 or less) and the time to achieve remission during the 5 weeks of treatment and 5-month follow-up after the light treatment [8].

### 4.4. Statistical Analysis

The data in the current study were analyzed on an intention-to-treat basis. Chi-square tests and *t*-tests were employed to compare the baseline demographic and clinical characteristics between the dropouts and the completers. Logistic regression analysis was performed, with dropout as the dependent variable. As this study aimed to investigate whether early symptom improvement could affect dropout, subjects who dropped out prior to the assessment at the end of the first week were excluded from the logistic regression analysis (N = 2). The independent variables selected for the present analysis included variables with *p* < 0.1 in the univariate analysis. Potential confounders (age, gender, treatment allocation, patient expectation, treatment-emergent adverse events) were included as independent variables in the adjusted model. The adjusted odds ratios (ORs) and 95% confidence intervals (CIs) are presented for the independent variables. Two-sided tests were used and a *p*-value of less than 0.05 was considered statistically significant. The data were analyzed using the Statistical Package System for Windows v25.0 (SPSS, IBM Corp., Chicago, IL, USA).

## 5. Conclusions

This study found that adherence to light therapy of 80% or less of the duration prescribed in the first week of treatment predicted a five-fold increase in the risk of dropout during the light treatment phase irrespective of patient expectations, adverse events, and treatment group. Future trials should enhance light therapy adherence monitoring by introducing objective measurements and should incorporate strategies to enhance adherence and treatment retention.

## Figures and Tables

**Table 1:** Baseline demographics and clinical characteristics of the Dropout group versus the Completer group (means ± standard deviations).

	Completers N = 74	Dropout N = 19	*p*-Value
**Demographics**			
Age	46.6 ± 11.6	45.8 ± 12.3	0.79
Gender, Female, N (%)	58 (78)	16 (84)	0.57
Education, N (%)			0.64
Primary	8 (11)	3 (16)	
Secondary	43 (58)	12 (63)	
Tertiary	23 (31)	4 (21)	
Employment, N (%)			0.14
Unemployed	25 (34)	4 (21)	
Housewife	19 (26)	9 (47)	
Part-time	10 (13)	4 (21)	
Full-time	20 (27)	2 (11)	
Living status, N (%)			0.54
Alone	12 (16)	2 (10)	
With others	62 (84)	17 (90)	
Monthly income in HKD ^^^, N (%)			0.62
<10,000	28 (38)	5 (26)	
10,001–20,000	21 (28)	7 (37)	
>20,001	25 (34)	7 (37)	
Season of enrollment, N (%)			0.48
Spring/Summer	34 (46)	7 (37)	
Autumn/Winter	40 (54)	12 (63)	
Expectation score	60.3 ± 20.3	65.0 ± 15.7	0.35
**Sleep diary parameters**			
Bedtime	01:16 ± 01:45	01:42 ± 01:58	0.37
Time to fall sleep	01:51 ± 01:39	02:27 ± 01:52	0.18
Wake time	09:23 ± 02:14	10:11 ± 02:40	0.20
Rise time	10:13 ± 01:54	11:12 ± 02:27	0.066
WASO	00:32 ± 00:37	00:35 ± 00:44	0.76
Sleep midpoint	05:37 ± 01:48	06:19 ± 02:11	0.16
Time in bed	8:56 ± 1:36	9:26 ± 1:52	0.26
**Clinical characteristics**			
Duration of illness, yr	12.9 ± 10.1	17.0 ± 13.9	0.19
17-HDS	19.0 ± 7.0	20.3 ± 5.9	0.48
Atypical score	5.7 ± 3.5	5.9 ± 3.4	0.82
HAMA	21.4 ± 11.0	23.1 ± 9.8	0.53
HADS	21.2 ± 5.7	23.2 ± 8.1	0.34
ISI	17.6 ± 5.6	16.3 ± 7.5	0.44
SF 36	285.9 ± 107.1	287.6 ± 88.2	0.95
BSSI	11.8 ± 6.4	10.7 ± 5.9	0.50
CFS	20.6 ± 6.5	18.9 ± 8.3	0.35
YMRS	0.85 ± 1.6	0.68 ± 1.1	0.66
MEQ	33.6 ± 5.4	32.6 ± 7.2	0.56
Extreme evening, N (%)	23 (31)	8 (42)	0.36

^^^ USD 1 = HKD 7.8. WASO: Wake after sleep onset; 17-HDS: 17-Item Hamilton Depression Scale; HAMA: Hamilton Anxiety Scale; HADS: Hospital Anxiety and Depression Scale; ISI: Insomnia Severity Index; SF36: Short-Form 36-Item Health Survey; BSSI: Beck’s Scale for Suicide Ideation; CFS: Chalder Fatigue Scale; YMRS: Young Mania Rating Scale; MEQ: Morningness–Eveningness Questionnaire; Extreme eveningness: defined as an MEQ score of 30 or below.

**Table 2:** Light treatment allocation and the prescription of psychotropic medications for the Dropout and Completer groups.

	Completers N = 74	Dropout N = 19	*p*-Value
**Treatment, N (%)**			
Dim red light	34 (46)	12 (63)	0.41
Bright light therapy	40 (54)	7 (37)	
**Prescription Pattern**			
Antidepressants, N (%)	58 (78)	14 (74)	0.76 ^#^
Antipsychotics, N (%)	16 (22)	7 (37)	0.17
Benzodiazepine, N (%)	32 (43)	8 (42)	0.93
Hypnotics, N (%)	21 (28)	4 (21)	0.52
Mood stabilizers, N (%)	6 (8)	2 (10)	0.66 ^#^

^#^ Fisher’s exact test.

**Table 3:** Treatment adherence and early changes in clinical outcomes from baseline to week 1.

	Completers N = 74	Dropout N = 17	*p*-Value
**Week 1 treatment**			
Prescribed LT start time, HH:MM ± SD	10:25 ± 02:20	11:16 ± 02:25	0.21
LT start time recorded by sleep diary, HH:MM ± SD	10:27 ± 02:11	11:32 ± 02:27	0.11
^a^ Duration of LT, min ± SD	174 ± 54	110 ± 82	0.001
^b^ LT duration < 80%, N (%)	28 (38)	13 (77)	0.004
TEAE, N (%)	25 (34)	7 (41)	0.57
Changes in mean scores from baseline *			
17-HDS	−2.9 ± 6.1	−3.3 ± 6.9	0.82
Atypical score	−1.0 ± 3.2	−0.9 ± 3.7	0.92
HAMA	−2.4 ± 8.3	−3.1 ± 11.4	0.77
HADS	−0.7 ± 4.3	−1.1 ± 3.4	0.69
ISI	-0.3 ± 3.5	−0.9 ± 2.6	0.54
BSSI	−0.9 ± 4.8	−2.6 ± 6.8	0.23
CFS	−2.1 ± 5.7	−1.5 ± 6.1	0.71
YMRS	−0.2 ± 1.8	0.8 ± 2.1	0.046
MEQ	2.4 ± 5.4	2.8 ± 4.3	0.74

* Negative value = reduction of week 1 score as compared to the baseline score. LT: Light therapy; SD: Sleep diary; TEAE: Treatment-emergent adverse events, which was defined as a change from baseline to at least moderate severity at the week 1 follow-up. ^a^ Duration of LT = the weekly total duration of light therapy performed, as recorded by the treatment log. ^b^ LT duration < 80% = receiving less than 80% of the prescribed duration of light therapy (e.g., <168 min, as total duration of light therapy prescribed was 30 min × 7 days = 210 min per week).

**Table 4:** Logistic regression analysis for the predictors of dropout.

	Model 1			Model 2		
Independent Variables	*p*-Value	Odds Ratio	CI	*p*-Value	Odds Ratio	CI
LT duration < 80%	0.017	4.68	1.32–16.66	0.015	5.85	1.41–24.41

CI: Confidence interval. Model 1 adjusted for rise time at the baseline, change in YMRS at week 1, and LT duration <80%. Model 2: adjusted for the factors in Model 1, age, gender, light treatment, patient expectation, and treatment-emergent adverse events at week 1.

## Data Availability

Reasonable requests to access the related data will be considered on a case-by-case basis and should be made to the corresponding author. Ethical approval for data-sharing agreements is required to share data in order to protect participant confidentiality.
